# Supplementary material for: Individual and community-level determinants, and spatial distribution of institutional delivery in Ethiopia, 2016: Spatial and multilevel analysis
Source: PLoS One. 2020 Nov 12;15(11):e0242242. doi: 10.1371/journal.pone.0242242 (PMC7660564; doi:10.1371/journal.pone.0242242)
Supplement: Attachment — Submitted filename: Reviewed Comments on the PLOSONE Manuscript-Latest.docx [file pone.0242242.s001.docx]

Additional Comments on the paper Titled: “***Individual and community-level determinants and spatial distribution of institutional delivery in Ethiopia, 2016: Spatial and multilevel analysis***”

**On Page 2**;

Line 31: “EDHS”- needs to be defined in full the first time of introduction and put the abbreviation in parenthesis. Then, you can proceed on to use the acronyms subsequently in the text.

Lines 39-48

Observations:

1. It is conventional to always state the p-value and the z-score whenever, Moran’s I is computed. The z-score value is key to decision making and interpretation of your estimate result.
2. I advise the authors try to present the confidence intervals alongside the ‘Adjusted Odd ratios’ estimates

Page 5

Line 94: the ‘**Some** indicator’…… should read: “**One** indicator”…..

Line 95: What is the purpose of the letter ‘R’ after 2016?

**Page 6**

Line 123: a period (.) is required after ‘most populous country’. You also need either “namely” or hyphen (-) **systems**, and **Primary**

**Line 126: the sentence: ‘**The number of hospitals varies from region to region ~~in response to differences in~~ population size**’** could be reworded as **“**The number of hospitals varies from region to region **depending on the** population size.

**Line 127:** after ‘population size,’ put semicolon **(;)** continue the sentence as**: “**for example the most populous region, Oromia has 30 hospitals.”

**Line 129: before** ‘fourth’ an article **“the”** is missing. A period (.) is missing after ’16 hospitals’

Line 132**:** EAs need to be defined in full for the first time

Line 141: Article “the” is missing after the word ‘all’ and before the word ‘average’ (2 places)

Page 7; line 151: close the parenthesis after the phrase-‘health post’, before the period (.)

**Page 8**

**Line 173:** I suggest replace ‘based on’ with **“using”** and the phrase **‘**by accessing’ with “**accessed**”. Write the acronyms DHS in full for the first time please.

**Lines 181 to 182:** The sentence ‘The questionnaires were pretested in all three local

languages (Amharic, Oromia, and Tigrigna) to make sure’….. Be re-worded as: “The questionnaires were pretested in all **the** three **major** local languages (Amharic, Oromia, and Tigrigna), in Ethopia to ensure”……..

Line 191: A comma (,) is missing between regression and which; “regression, which”

Line 195: Replace ‘based on’ with “using”

**Page 9**

**Lines 205-209:** Is there any difference between the two highlighted parameters (or symbols)? If none, please be consistent. You right symbol for sigma square (if that is what you want), could be obtain from **Equation Editor**

Also, use Equation Editor to present the formula:

**Line 214:** Instead of saying**: ‘**empty model’ why not either say**: “null model” or “intercept only model”?**

**Line 219:** What justification have you for a p-value $\boldsymbol{\leq}$ **0.2? Explain…**

**Page 10**

**Line 227**: I suggest your subtitle should read**: “Spatial Autocorrelation Analysis”**

**Lines 231-232:** why using **‘disease’** while illustrating your **Moran’s I** possible range value instead of **‘**institutional delivery’**?** Please, be consistent**!**

‘Moran's I Values close to −1 indicate ~~disease~~ (or institutional delivery centres?) dispersed, whereas Moran's I close to +1 indicate ~~disease~~ (or institutional delivery centres?) clustered and disease’

**Line 237-238:** Replace this section of your sentence**: ‘**areas of the country to know the burden of a certain event’ **with “**areas of the country aimed at investigating/identifying impacts of” a certain event

**Line 243: Thus,** is missing before **‘**Ordinary Kriging spatial……**’**

**Page 11**

**Lines 266-271.** Fix the paragraph accordingly as presented:

Among 11,022 women who gave birth within five years preceding the survey participated, 4,851 (44.0%) were Oromia and 0.24% (N=26) in the Harari region. The majority, 9,807 (89.0%) of the participants were rural residents. About two-thirds (66.7%) and a half (47.8%) of the women and husbands had no formal education, respectively. Regarding the age of the participants,

71.8% (N=7914) were in the age group 20 -34 years. The majority of the respondents with proportional frequency of about 41.4% (N=4,564) were Muslim followers

**Page 12**

**Line 287-288:** Could authors try and confirm if there is any form of contradictions between the following lines of statements?

**‘….w**ith the low rate of institutional delivery cases which were located in Addis Ababa, Dire Dawa and Tigray region’	**and**

**Line 299-300:**

**‘…..**the highest institutional delivery was detected in Addis Ababa, Dire Dawa, Harari, central Gambella and Tigray regions’

**Page 13**

**Line 309**: “…… women from two different clusters, ~~of~~ women within ~~at~~ the cluster with a higher likelihood of having…”

**Lines 310**-311

“……having institutional delivery than ~~as compared~~ with women within ~~at~~ cluster”

**Lines 312-313**: Restructure the following sentence:

‘For model comparison deviance was used. The final model was the best-fitted model since it had the lowest deviance’

**Line 320**: the error in the below expression has been fixed for you. I suggest the authors cross-check the similar expressions and fix any spotted error.

(AOR: 0.48; 95%CI: 0.32-0.74)

Line 337: the word “women” is missing here:

“…..compared to women residing in pastoral regions”

Page 14

Line 340: Restructure the expression:

‘………….times high likely to have institutional delivery as compared to rural residents…’

I suggest you replace with the following expression:

“………..times higher likelihood of having institutional delivery than rural residents…”

Line 352: Is the word ‘introduced’ in the following expression necessary?

‘……. skilled health personnel **introduced** and….’

Line 363: I assume the following expression: ‘……regression analysis exhibited maternal….’ reads better by re-wording as follows: “….regression analysis showed/revealed that maternal…’

Line 365: I assume the authors wanted to say:

“Education key to adapting positive behaviour….” Rather than: ‘Education is thought a key to adapt positive behavior’?

Line 367: I assume the following expression:

‘…….education more likely attended health facilities for delivery services because they know the advantage than those who did not attain formal education.’

reads better as follows:

“……education are more likely to attend health facilities for delivery services because they are well informed of the advantage/benefit than those without any formal education.”

Page 15

Line 372: Fix the error in the following expression: ‘……….electronic and print~~ed~~ media….’

Line 377: ‘Ethiop’ or “Ethopia”?

Lines 397-398: ‘…..supposed capable to cover the….’

Page 17

Line 435: I suggest you replace the word ‘little’ with “sparingly” (or scantily)
