# Supplementary material for: Individual and community-level determinants, and spatial distribution of institutional delivery in Ethiopia, 2016: Spatial and multilevel analysis
Source: PLoS One. 2020 Nov 12;15(11):e0242242. doi: 10.1371/journal.pone.0242242 (PMC7660564; doi:10.1371/journal.pone.0242242)
Supplement: Attachment — Submitted filename: Point by point response.docx [file pone.0242242.s002.docx]

**PLOS ONE**

**Point by point response for editors/reviewers comments**

**Manuscript title:** Individual and community-level determinants and spatial distribution of institutional delivery in Ethiopia, 2016: Spatial and multilevel analysis

**Manuscript ID:**  PONE-D-19-33251

**Dear editor/reviewer.**

Dear all,

We would like to thank you for this constructive, building, and improvable comments on this manuscript that would improve the substance and content of the manuscript. We considered each comment and reviewers on the manuscript thoroughly. Our point-by-point responses for each comment and questions are described in detail on the following pages.

**Response to editors comments**

1. Some similar studies have discussed this issue in the same country before. See, for example, Mekonnen, Z.A., Lerebo, W.T., Gebrehiwot, T.G. et al. Multilevel analysis of individual and community-level factors associated with institutional delivery in Ethiopia. BMC Res Notes 8, 376 (2015). <https://doi.org/10.1186/s13104-015-1343-1>; Mezmur M, Navaneetham K, Letamo G, Bariagaber H (2017) Individual, household and contextual factors associated with skilled delivery care in Ethiopia: Evidence from Ethiopian demographic and health surveys. PLoS ONE 12(9): e0184688. <https://doi.org/10.1371/journal.pone.0184688>.
a) So what this study added to the current knowledge?

**Authors’ response:** Thank you editor for the concerns. These two previous studies were conducted based on EDHS 2005 and 2011 data to investigate the individual and community level determinants of institutional delivery using multilevel analysis. But both studies are failed to capture the spatial distribution of institutional delivery using ArcGIS and SaTScan analysis to identify the significant hotspot areas where institutional delivery was low even if studies done on the prevalence of institutional delivery in different parts of Ethiopia revealed that the prevalence has been varied across the country. Besides, in this study, the data were not weighted even if the DHS statistician recommended using weighted data based on strata, PSU, and weighting variables to restore the representativeness as well as to get reliable standard error and estimate. Therefore, we investigated the individual and community level determinants and spatial distribution of institutional delivery in Ethiopia based on the most recent EDHS data. Thus, exploring the spatial distribution of institutional delivery is important to identify significant primary and secondary hotspot areas where institutional delivery is low, this could help to design targeted public health interventions to the identified hotspot areas to enhance health facility delivery to reduce preventable maternal and newborn mortality. Furthermore, hence the data we used were weighted the estimates are reliable. (see the Backroung section, line 95-106, page 6)

b) A further comparisons with the above studies need to be included in the discussion section.

**Authors’ response**: Thank you editor for the comments. We had incorporated it. (see the revised document)

c) Also, the literature review needs to be more robust and to include all the similar that tackled this issue in Ethiopia

**Authors’ response**: Thank you, editor. We had incorporated previous study findings conducted on institutional delivery by extensively searching literature. (See the revised manuscript)

2. It was not mentioned why the manuscript has focused on this only or this period only? Was there any inclusion/exclusion criteria considered?

**Authors’ response**: Thank you editor for the concerns. We have used the EDHS 2016 data for this study since this Survey is the most recent in Ethiopia. For this study, we excluded the respondents where the outcome variable (place of delivery) was missed and for the spatial analysis, we excluded those women in the Enumeration Areas (EAs) with zero latitudes and longitude.

3. Neither strength nor the limitations of this study were mentioned?

**Authors’ response**: Thank you editor, we have included strength and limitations of the study in the revised manuscript. (See the Strengths and limitation section, line 427-438, page 21)

4. The future work plan was not mentioned and how we plan to overcome the limitations of this study in the future.

Authors’ response: Thank you editor, we have incorporated in the revised manuscript. (See the revised manuscript, Conclusion section, line 448-452, page 22)

5. We note that Figures 1 and 4-6 in your submission contain map images which may be copyrighted. All PLOS content is published under the Creative Commons Attribution License (CC BY 4.0), which means that the manuscript, images, and Supporting Information files will be freely available online, and any third party is permitted to access, download, copy, distribute, and use these materials in any way, even commercially, with proper attribution. For these reasons, we cannot publish previously copyrighted maps or satellite images created using proprietary data, such as Google software (Google Maps, Street View, and Earth). For more information, see our copyright guidelines: http://journals.plos.org/plosone/s/licenses-and-copyright.

**Authors’ response**: Thank you editor for the concern. The map is not copyrighted rather we have done using ArcGIS and SaTScan software based on the shapefile of Ethiopia received from Ethiopian Central Statistical Agency (CSA) by explaining the purpose of the study and GPS data (longitude and latitude) from measure DHS program by explaining the objective of the study through online requesting and allow us to access the shapefile and GPS data. Now we cite the source of the shapefile since it is needed to explore the spatial distribution of institutional delivery. Therefore, the maps presented in our study are not copyrighted rather it was our spatial analysis result.

**Response to reviewers comments**

**Reviewer#1**

1. The research focus though not new, it has helped to fill some of the identified gaps in the existing direction of the study.
   The authors have demonstrated good understanding of the subject and had written the manuscript professionally and intelligently. Efforts put into the writing the manuscripts and the analysis have shown clearly that the authors are skilful and experienced researchers. The methodology adopted in the data analysis fits appropriately to the nature of the data and in fulfilment of the aim/objectives of the study. However, clearer research question(s) need to be established. The authors would need to consult experienced language editor to enhance the flow of the manuscripts.

**Authors’ response**: Thank you, reviewer, for the comments. The research questions in our study were, 1) to identify the individual and community level factors associated with institutional delivery, 2) to explore the spatial distribution of institutional delivery, and to identify significant hotspot areas where institutional delivery utilization is low to design targeted public health interventions. We had extensively edited and modified the entire document. (see the revised manuscript)

2. Some minor errors/concerns need to be addressed. I have chronicle some the concerns I feel the authors need to address to enhance the quality of the paper and to meet the required standard of the intended journal

**Authors’ response**: We thank the reviewer for your great effort for the betterment of our work. We accepted your comments and modified accordingly. (see the revised manuscript)

**Reviewer# 2**

1. this paper presents an interesting approach to understanding variation in institutional delivery in Ethiopia using both multilevel modeling and GIS. While the modeling strategy is interesting, the paper would be improved if the authors spent more time discussing the two approaches (the multilevel modeling and the GIS approach) in tandem and how they build on one another. More synthesis on the use of the added value and joint examination of the results would be interesting and would strengthen the paper.

**Authors’ response**: Thank you, reviewer. We synthesize both the use of spatial and multilevel modeling and for further we cite references. (See the revised manuscript)

2. The authors will need to carefully proof read the manuscript. In some places, it is difficult to follow because of grammatical errors. It may help to have an outside person edit the document for grammar. I’ve pulled a few examples here: page 1 line 63: “For example, the estimated 130,000 maternal deaths happened in 2017 in those countries [3].” Page 1 lines 71-72, “plentiful numbers of women in developing countries give birth at homes.” Page 2 line 90: “So far, Ethiopia has made a lot to curb maternal...” • Page 1 line 67: I think it would be more accurate to say that strengthening facility delivery or skilled attendance would “reduce” not “alleviate” the burden of preventable maternal death. • Page 2, line 96: is the low facility attendance due to limited health facilities, bad infrastructure, etc?

**Authors’ response**: Thank you, reviewer, for the comments. we had extensively edited the whole document with the help of language experts at university of Gondar. (See the revised manuscript)

3. There is a lot of background information provided in the “study design, setting and period” section. The authors may want to consider integrating some of the background about Ethiopia into the background section, as it is somewhat difficult to follow in the methods section. The authors should indicate that they are using DHS data and describe it from the beginning of the methods section when they first discuss the survey. Line 151: missing closed parentheses

**Authors’ response**: Thank you, reviewer. we have modified it. (see the revised manuscript)

4. The authors may want to consider including the general equation that they use. It is somewhat confusing for the authors to describe the outcome variable as Yi given that multilevel models usually require a nested structure, so that individual i is usually nested in community j and so forth. Also, how is region a community level variable? Is that not a third level of the model? Or, are the authors including dummies for region as fixed effects?

**Authors’ response**: Thank you, reviewer. we incorporated the general equation of the multilevel model we fitted. We use Enumeration area/clusters as a random effect as level two since women within the selected enumeration area are more correlated than the women from different clusters. In EDHS except for region, distance to the health facility, and residence the other variables were collected at the individual level. We tried to consider the region as the third level of the model but the result was the same as two level multilevel model and besides, we didn't get any variable collected at the region level. We categorized the region into four groups as Pastoral, Semi pastoral, City administration, and Agrarian in the multilevel analysis and considered as a community level variable at level 2.

5. The authors should more clearly specify how they defined the level 2 community variables. As it stands, it is unclear how the authors measured community level media exposure. It would also be helpful for them to cite other papers that have used EA in DHS as a proxy for community.

**Authors’ response**: Thank you, reviewer. We included how we generated community-level variables. In EDHS data except for region, residence, and distance to health facility these variables were collected at individual levels. The EDHS data has hierarchical nature means women are nested within-cluster therefore we want to assess the individual and cluster level variables that affect institutional delivery. Then to assess whether community media exposure has a significant effect on institutional delivery we aggregated media exposure collected at the individual level to community level/cluster level. Then we categorized as high community media exposure and low community media exposure based on the national median value since it was not normally distributed. (See the revised manuscript)

6. The calculation of the PCV in binary multilevel models is not straightforward due to the level-1 variation of the binary model. Can the authors describe in full what approach they used?

Authors’ response: thank you, reviewer, for the comments. The proportional change in variance (PCV) measures the total variation attributed by individual-level factors and area-level factors in the multilevel model. PCV is used to show the total variability explained by the final model (model with individual and community level variable simultaneously) relative to the null model, it is like the coefficient of determination (R^2^) in the linear regression model. It is interpreted as the total variability of institutional delivery explained by the final model. As you know in the multilevel binary logistic regression model the level-1 variation is constant (π^2^/3) unlike the linear regression since there are no residuals. Therefore, we calculated by substracted the variance of institutional delivery in the final model from the variance in the null mode divided by the variance in the null model.

7. The authors may want to consider condensing some of the material and reorganizing some of the subheadings in the methods section. For example, the data collection procedure section could be combined with the description of the survey. As of now, some of the information is duplicative and the section is somewhat difficult to follow.

**Authors’ response**: Thank you, reviewer, we organized to combined related subheadings together. (See the revised manuscript)

8. What statistical program did the authors use and using what estimation technique?

**Authors’ response**: Thank you reviewer. For analysis we used melogit STATA command (melogit institutional_delivery i.ANC i.region i.parity i.residence i.communty_media_exposure i.maternal_education i.materna_age i.husband_education i.birth_interval i.wealth_status [pw=wgt] || v001:, or), using maximum likelihood estimation technique. To calculate the MOR and ICC we install the xtmrho stata command and run after running the full model.

9. Results
   • Does the fact that DHS throws the spatial coordinates of GPS influence to ensure that the exact location of the clusters is not revealed influence the results in any way? In particular, page 11 (the section beginning at line 292)?

**Authors’ response**: thank you, reviewer, for the comments. In EDHS as GPS data (latitude and longitude) taken in the Enumeration area level were displaced up to 5km in Urban area, and up to 10 km in rural areas because of privacy issues, therefore, this could bias our findings and we acknowledge in the limitation sections of the study. (See the revised manuscript, limitation section, line 434-436, page 21)

10. The interpretation of the odds ratio from the null model is confusing (line 305). I assume that 7.01 refers to the intercept? The authors may want to ensure that their interpretation is correct, and if so, explain it in a way that is more clear.

Authors' response: Thank you, reviewer. This MOR result in the null model. MOR quantifies the variation between clusters (the second level variations) by comparing two women from two randomly chosen, different clusters. The result was MOR= 7.01, 95% CI: 6.02, 9.17 it indicates that if we randomly select two women from two different clusters. A woman from the cluster with a high likelihood of institutional had 7.01 times higher odds of having institutional delivery compared with women form clusters with lower institutional delivery. Therefore, this finding was not the intercept rather it is another method of assessing heterogeneity across clusters like ICC.

11. The authors state that “About 73 percent of the variability in institutional delivery was explained by the full model.” Is this the model overall? What about the different levels?

**Authors’ response**: Thank you, reviewer, for the concern. PCV is used to measure the total variation in institutional delivery explained by the final model (a model with both individual and community-level factors simultaneously) in relative to the null model ( Model without independent variables).

Therefore, about 73% of the total variation in institutional delivery was explained by the overall model.

12. In the methods section, the authors describe “region” as a community-level variable. Does region refer to urban/rural as indicated in the results section?

Authors' response: Thank you, reviewer, for the comments. In Ethiopia, there are 9 regions and 2 city administrations. In our study, we categorized the region into 4 groups. 1, pastoralist region (Benishangul, Somali, Gambella, and Afar), Semipastorlaist (Oromia, SNNPR), Agrarian (Amhara and Tigray) and City administration (Addis Ababa, Dire Dawa, and Harari) based on literature. Rural and Urban were for the variable residence.

13. The authors may want to discuss community level variables separately from individual level variables as it is somewhat confusing to understand the results.

Authors' response: Thank you, reviewer. we have discussed it separately, first, we discussed the individual variables significantly associated with institutional delivery. (See the Discussion section, line 347-425, page 17-21)

14. Discussion

• An important piece missing from the discussion is a synthesis of the results and a discussion of why context matters. The authors do a good job of summarizing the results and then discussing them in light of other literature, but it would be interesting for the authors to focus on understanding the combined results from both analytical approaches to deepen the understanding of the role of community context in Ethiopia.

Authors' response: Thank you, reviewer. we have written out in the revised manuscript.

15. • The authors do not discuss the results of the very interesting GIS analysis in the discussion.

Authors' response: Thank you, reviewer. we have discussed it. (See the Discussion section, line 361-370, page 18)
