# Supplementary material for: Individual and community-level determinants, and spatial distribution of institutional delivery in Ethiopia, 2016: Spatial and multilevel analysis
Source: PLoS One. 2020 Nov 12;15(11):e0242242. doi: 10.1371/journal.pone.0242242 (PMC7660564; doi:10.1371/journal.pone.0242242)
Supplement: Attachment — Submitted filename: Point by point response.docx [file pone.0242242.s003.docx]

**Point by point response for editors/reviewers comments**

**Manuscript title:** Individual and community-level determinants, and spatial distribution of institutional delivery in Ethiopia, 2016: Spatial and multilevel analysis

**Manuscript ID:**  PONE-D-19-33251R1

**Dear editor/reviewer.**

Dear all,

We would like to thank you for this constructive, building, and improvable comments on this manuscript that would improve the substance and content of the manuscript. We considered each comment and clarification questions of editors and reviewers on the manuscript thoroughly. Our point-by-point responses for each comment and questions are described in detail on the following pages. Further, the details of changes were shown by track changes in the supplementary document attached.

**Response to reviewers comment**

**Reviewer # 2**

1. General

There are still some grammatical issues that the authors should address prior to publication. In particular, there are still grammatical mistakes, misplaced words, and incorrect/inconsistent use of tense.

**Authors’ response**: Thank you reviewer for the comments. We extensively edited the whole document for the grammatical error, typical errors, and sentence structure with the help of language experts at UOG. (See the revised manuscript)

2. Methods
   2.1. The organization of the methods section is still somewhat confusing. The multilevel equation specified by the authors appears under the outcome variable description when it seems like the discussion of the model would be more appropriate under the description of multilevel analysis that appears later in the methods section.

**Authors’ response**: Thank you reviewer for raising the important comments. We accept your comments and we placed the equation in the multilevel analysis section. (See the Method section, line 191- 202, page 10)

2.2. . The authors describe their calculation of the PCV as being reliant on calculation the total variance. The authors should include how they calculate the total variance, given that the VPC is more difficult to calculate in binary-response models due to the different scale of the level 1 variance because of use of the link function.

**Authors’ response**: Thank you reviewer for the comments. As you stated, it is difficult to calculate VPC/ICC is more difficult since unlike the linear regression model the individual-level variance in logistic regression is assumed to follow the standard logistic distribution with mean 0 and variance of π^2^/3 (3.29). So, the individual level variance is constant that is π^2^/3, we add the cluster level variance with π^2^/3 to get the total variance in the null model and in the final model. Then we calculate PCV by using the formula (variance in the null model – variance in the final model)/variance in the null model.

2.3. Furthermore, the authors may want to discuss the implications of their choice of estimation technique (maximum likelihood) on the estimates of variance, given that maximum likelihood estimation tends to underestimate the variance at higher levels in multilevel binary models, and often MCMC estimation is used instead when the variance parameters are of substantive interest. The authors may want to consider adding this in the limitations section

**Authors’ response**: Thank you reviewer for the concerns. We used the maximum likelihood estimation technique to estimate the estimates of variance since we used the classical multilevel binary logistic regression and the sample is large. We plan to use MCMC to estimate the variance using MCMC but to do this it needs the posterior distribution (bayesian approach). And now we have checked by simulating the data and estimate the variance using MCMC but the value is the same as the MLE findings since the sample is adequate and the prevalence is 26.2.

2.4. Results, The authors calculate the total variance explained by the addition of the covariates in the model, but they do not decompose the variance to examine the variance explained at the community level. This would be helpful, and again goes back to my previous comment asking the authors about how they calculated the total variation in the model.

**Authors’ response**: Thank you reviewer for concerns. PCV is commonly done in multilevel analysis to examine by how much the full model explains the variations of institutional delivery as compared to the null model. And we calculate the PCV for three models (model1 model2, and model 3) in relative to the null model. Logically we need that the PCV value to be highest in the final model as the number of covariates incorporated is increased, based on these the final model was 73% indicates that the final model explains the variation in institutional delivery by 73%, it is like the coefficient of determination in the linear regression model. So, this 73% is explained by the mutual effects of the individual as well as community-level variables, and it is difficult to partition how much is explained by the individual and how much is by the community level variables. Just PCV is supportive, beyond that we have used deviance, MOR, ICC, and LR-test to check whether the model is improved or not. Just to know how much is attributable to the community level and how much is by community level we can get in model 1 and model 2 as we presented in the table.

3. Discussion
   1. The discussion still requires some grammatical editing and certain parts are difficult to follow. In particular, paragraph 2.

**Authors’ response**: Thank you the reviewer for the comments. We extensively modify it. (See the revised manuscript)

**Reviewer #3**

1. The authors have addressed concerned earlier. Having reviewed the current version of the manuscript I can confidently confirmed that the article has now significantly met the set conditions for it to be published in this journal.

Thus, I hereby recommend that the manuscript be accepted for publication. The only area I wish the authors should try and adjust is in the equations in lines 135-142. Using equation editor could help to fine-tune the notations/subscripts of the respective equation's parameters

**Authors’ response**: Thank you reviewer for the comments. We accept the comment and we put in equation form. (See the revised manuscript)
